# Vasodilative effects of prostaglandin E_1 _derivate on arteries of nerve roots in a canine model of a chronically compressed cauda equina

**DOI:** 10.1186/1471-2474-9-41

**Published:** 2008-04-08

**Authors:** Masayoshi Shirasaka, Bunji Takayama, Miho Sekiguchi, Shin-ichi Konno, Shin-ichi Kikuchi

**Affiliations:** 1 Department of Pharmacy, Fukushima Medical University Hospital, 1-Hikarigaoka, Fukushima City, Fukushima 960-1295, Japan; 2 Department of Orthopaedic Surgery, Fukushima Medical University School of Medicine, 1-Hikarigaoka, Fukushima City, Fukushima 960-1295, Japan

## Abstract

**Background:**

Reduction of blood flow is important in the induction of neurogenic intermittent claudication (NIC) in lumbar spinal canal stenosis. PGE_1 _improves the mean walking distance in patients with NIC type cauda equina compression. PGE_1 _derivate might be effective in dilating blood vessels and improving blood flow in nerve roots with chronically compressed cauda equina. The aim of this study was to assess whether PGE_1 _derivate has vasodilatory effects on both arteries and veins in a canine model of chronic cauda equina compression.

**Methods:**

Fourteen dogs were used in this study. A plastic balloon inflated to 10 mmHg was placed under the lamina of the 7th lumbar vertebra for 1 week. OP-1206-cyclodextrin clathrate (OP-1206-CD: prostaglandin E_1 _derivate) was administered orally. The blood vessels of the second or third sacral nerve root were identified using a specially designed surgical microscope equipped with a video camera. The diameter of the blood vessels was measured on video-recordings every 15 minutes until 90 minutes after the administration of the PGE_1 _derivate.

**Results:**

We observed seven arteries and seven veins. The diameter and blood flow of the arteries was significantly increased compared with the veins at both 60 and 75 minutes after administration of the PGE_1 _derivate (p < 0.05). Blood flow velocity did not change over 90 minutes in either the arteries or veins.

**Discussion:**

The PGE_1 _derivate improved blood flow in the arteries but did not induce blood stasis in the veins. Our results suggest that the PGE_1 _derivate might be a potential therapeutic agent, as it improved blood flow in the nerve roots in a canine model of chronic cauda equina compression.

## Background

Compression of the cauda equina by spinal stenosis is a major clinical problem associated with neurogenic intermittent claudication (NIC). A reduction in blood flow is considered an important factor in inducing NIC in lumbar spinal canal stenosis [1-5], and improving blood flow is expected to prevent NIC and leg symptoms. PGE_1 _leads to vasodilation in both arterioles and venules [6]. In patients with NIC type cauda equina compression, intravenous PGE_1 _has been shown to improve mean walking distance [1]. During myeloscopic observation of lumbar spinal canal stenosis, blood vessels on the cauda equina have been shown to dilate during NIC; thus microcirculatory disturbance of vessels on the cauda equina may play an important role in NIC [7]. In addition, as seen on myeloscopic examination, dilation of vessels is observed after administration of Lipo PGE_1 _in patients with lumbar spinal stenosis [8]. However, even if vasodilatory effects are achieved in both arteries and veins, it would lead to blood stasis, which may subsequently induce a reduction in blood flow. In the experimental study of the porcine cauda equina compression, blood flow in veins on spinal nerve is stopped by lower compression pressure than the one in arteries [4]. Therefore, it is important to investigate not only changes in the diameter of blood vessels but also changes in blood flow in both arteries and veins in the same model. The aim of this study was to assess the effect of a PGE_1 _derivate on nerve blood flow in both arteries and veins in a canine model of chronic cauda equina compression.

## Methods

A total of 14 dogs (average body weight 11.1 ± 0.4 kg) were used in this study. The experimental protocol was approved by the local animal ethics committee and conformed to Fukushima Medical University Guidelines, the Japanese Government Animal Protection and Management Law (No. 15), and the Japanese Government Notification on Feeding and Safekeeping of Animals (No. 6). All dogs were anesthetized with an intramuscular injection of 25 mg/kg ketamine hydrochloride (50 mg/ml Ketalar, Parke-Davis, Morris Plains, New Jersey) and 10 mg/kg pentobarbital sodium (50 mg/ml Nembutal, Abbott Laboratories, North Chicago, Illinois). After endotracheal intubation, anesthesia was maintained by inhalation of nitrous oxide (3 l/min), oxygen (3 l/min), and halothane (1%, SIC Chemicals Ltd, Bristol England).

### Chronic cauda equina compression model [9-12]

The dogs were placed prone, and a partial laminectomy of the caudal part of the sixth and seventh lumbar vertebras was performed. Compression balloons were made by welding thin polyethylene sheaths together. The width of the balloon was 20 mm. The balloon was folded into three layers and gently placed under the lamina of the seventh lumbar vertebra. An ATS-1000 compressed-air system (Aspen Laboratories, Littleton, Colorado) was used to infuse a substance called "konnyaku" into the balloon at a slow rate of 10 mmHg infusion pressure. This was based on a clinical study that showed that epidural pressure at the stenotic level is approximately 10 mmHg in spinal stenosis patients in the prone position [13]. Konnyaku, which is starch from the plant *Amorphophalus rivieri*, becomes liquid after being mixed with water. Water at room temperature was sufficient for konnyaku to become viscous, which occurred in approximately 10 minutes. There was no injury to the nerve tissue when the konnyaku in the balloon became viscous. When there was no further flow of konnyaku into the balloon, the infusion pressure was maintained for 30 minutes to compensate for the pressure loss caused by displacement of the tissues in the spinal canal. The diameter of each balloon exceeded that of the spinal canal and a reliable pressure transmission was provided to the cauda equina, as confirmed in separate calibration experiments. The inflated balloon, still under infusion, was then ligated at the carnial and caudal borders of the lamina of the seventh lumbar vertebra. Parts of the balloon located dorsally to the lamina were cut and removed. The balloon was then secured in place between the cauda equina and the lamina of the seventh lumbar vertebra.

One week after this procedure, 3 μg/kg with 10 ml normal saline OP-1206 OP-1206α-CD cyclodextrin clathrate (OP-1206α-CD), a prostaglandin E1 derivative, was administrated orally and the studies described below were performed. Dosing was based on the following findings of other studies: Ninety to 95% of OP-1206 a-CD is absorbed through the stomach in a rat, and the half-life of this drug is 7 hours [14]. There are no data regarding absorption rates in a canine model. In the clinical setting, oral therapy with 15 to 30 μg per day OP1206 a-CD is used for an adult patient. In rat studies [15,16], oral administration of 30–300 μg/kg OP-1206α-CD has been used; however, these concentrations are higher than those used in the clinical setting. In a rat model, the blood concentration of OP1206α-CD is approximately 2 to 2.5% of the total amount of drug administered. In a previous canine model, 3–30 ng/kg/min OP-1206α-CD was administered intravenously [17]. According to the clinical setting, 30 μg was chosen orally in this study. Because the mean weight of dogs in this study was 11.1 kg, **3 **μg/kg OP-1206α-CD was chosen as the best dose.

Studies were conducted 1 week after the initial operation for the model construction, applying the same surgical procedures (e.g., animal posture and anesthesia) as the initial operation.

**Figure 1:**
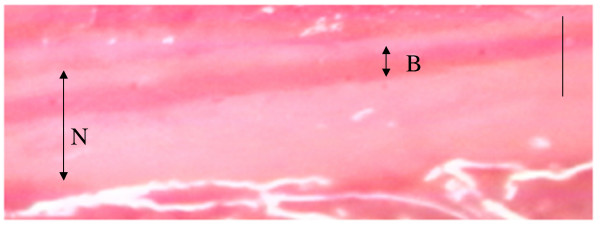
**The picture of nerve roots and blood vessels**. The blood vessel observed on the monitor. N: nerve root, B: blood vessel. Bar 100 μm.

**Figure 2:**
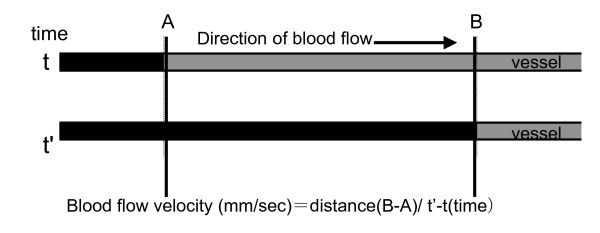
**Measurement of blood flow velocity**. Time (seconds) was measured using ink color changes (t'-t). The distance from A to B was 0.82 mm. The blood flow velocity (mm/sec) was calculated by the formula shown.

For application of OP-1206α-CD, a silicon tube was inserted and placed in the stomach. A catheter was inserted and placed in the left cervical artery for continuous monitoring of blood pressure. Another catheter, which was used for injection of ink, was inserted and placed in the abdominal aorta from the right femoral artery. In the preliminary experimental test, we had investigated the relationship among the location of the catheter tip, the branch of the lumbar artery, and the ribs using photofluorography. This test showed that the catheter was between the first and third lumbar arteries when its tip was placed on the same level as the distal bone edge of the twelfth rib. Therefore, the catheter tip was placed around the branch between the first and third lumbar arteries in this study. After insertion of the catheters, the dogs were placed prone. The lamina of the first and second sacral vertebras, and ligamentum flavum between the laminas of the seventh lumbar and first sacral vertebras were removed, and the cauda equina was exposed. The second or third sacral nerve root was identified just caudal to the compression site. The blood vessels were observed using a microscope equipped with a video-camera (Digital HI-SCOPE Video System, Hirox Co. Ltd., Tokyo, Japan) at 400× magnification. From the catheter in the abdominal aorta, 3 ml of blue ink (Pilot Co. Ltd., Tokyo, Japan) was injected manually for 1 second. When the ink flowed through the observed blood vessel, the color of the vessel changed from red to blue for several seconds. After checking the flow of ink from the catheter to the observed blood vessels, we started to record the blood vessels before and until 90 minutes after oral injection of OP-1206α-CD. Before and every 15 minutes until 90 minutes after administration of the drug, 3 ml of blue ink was injected through the catheter. In the preliminary test, blue ink flowed less than 5 seconds through an artery and longer than 5 seconds through a vein. Therefore, the blood vessels were divided into an artery and a vein according to phases of ink appearance. After these procedures, the measurements of diameter, blood flow velocity, and blood flow volume index in observed blood vessels were performed by an investigator in a blinded fashion using video recordings. The diameter of the blood vessels (μm) was measured three times for each ink injection using a monitor-connected video system equipped with a distance measuring device (Fig. 1). Blood flow velocity (millimeters per second) and time (seconds) of the color changes from red to blue were calculated using iMovie (Macintosh version) installed in a computer (Fig. 2). Blood flow volume index was defined as (1/2 diameter)^2 ^×(the blood flow velocity), to assess the changes in blood flow volume for each observed vessels [9,18,19]. The values of diameter and blood flow volume index before injecting OP-1206α-CD served as baseline values (100%). All recordings of diameter and blood flow volume index were expressed as a percentage of baseline values.

### Statistical analysis

Comparisons of the difference in diameters of the blood vessels and blood flow index among each group were performed by repeated measure ANOVA. P values of less than 0.05 were considered significant. Intra-observer reliability (R) was evaluated by one-way ANOVA. R values of more than 0.8 were considered to be "good" and more than 0.9 be "excellent". The R value was more than 0.9 in all groups; thus, the average data of each time point was used for the graphs.

## Results

Throughout the observation period, neither paralysis nor bladder dysfunction was observed in any dog. There was no wound infection. At the second operation, all balloons were found to be intact in the inserted position. The blood pressure did not change significantly before or after administration of OP-1206α-CD. A total of 14 blood vessels were observed (7 arteries and 7 veins).

### 1) Diameter of blood vessels

**Figure 3:**
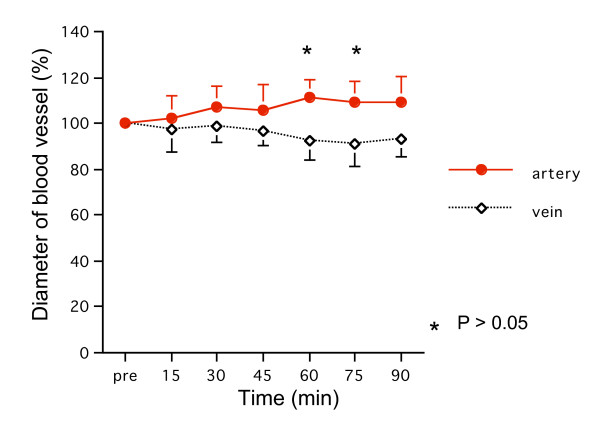
**Changes of the diameter**. There was a significant difference in the diameter of arteries compared with veins at 60 and 75 minutes after administration of OP-1206α-CD (p < 0.05).

The diameter of arteries, but not veins, dilated gradually. There was a significant difference in the diameter of the arteries at 60 and 75 minutes after administration of OP-1206α-CD compared with the diameter of the veins (p < 0.05) (Fig. 3).

### 2) Blood flow velocity

**Figure 4:**
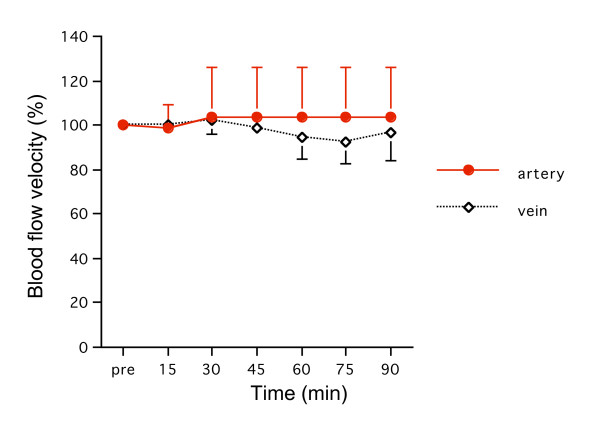
**Changes of blood flow velocity**. There was no significant difference in arteries and veins in blood flow velocity over 90 minutes.

Blood flow velocity did not change after administration of OP-1206α-CD (Fig. 4). There was no significant difference between arteries and veins in velocity for 90 minutes.

### 3) Blood flow index

**Figure 5:**
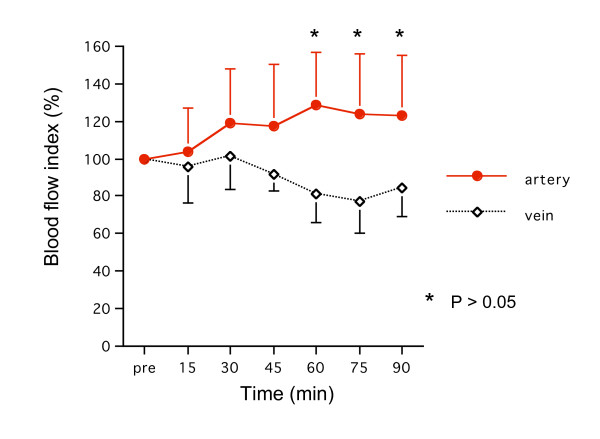
**Changes of blood flow index**. The blood flow index in the arteries increased significantly at 60, 75, and 90 minutes compared with the veins (p < 0.05).

The blood flow index in the arteries but not the veins was increased over 90 minutes with a significant difference between groups at 60, 75, and 90 minutes (p < 0.05) (Fig. 5).

## Discussion

NIC is a characteristic symptom among patients with lumbar spinal canal stenosis. It is aggravated by walking and leads to reductions in walking distance. Reduced intraneural blood flow is one cause of NIC [3,7]. Administration of PGE_1 _derivate and calcitonin, which are thought to improve blood flow, has been reported to improve walking distance of patients with NIC [1,20]. Intravenous administration of PGE_1 _increases nerve root blood flow velocity after lumbar diskectomy in spinal stenosis patients [21]. Experimental studies of PGE_1 _treatments have also been reported. Compression of cauda equina reduced blood flow in spinal nerve roots [4,5] and PGE_1 _increased blood flow and prevented the reduction in nerve conduction velocity in acute cauda equina compression [17]. In addition, intravenous injection of PGE_1 _derivative increased blood flow in chronic cauda equina compression [9]. In this study, the diameter of the arteries, but not the veins, increased after administration of PGE_1 _derivative under cauda equina compression. In addition, blood flow in the arteries increased after administration of PGE_1 _derivative. These results suggest that the PGE_1 _derivative has a vasodilatory effect and increases blood flow in arteries. No changes in blood pressure were observed following administration of the PGE_1 _derivate, and blood flow velocity was maintained during vasodilation. These findings indicate that the vasodilatory effect of the PGE_1 _derivate on arteries enables increased blood flow without inducing blood stasis. In veins, the PGE_1 _derivate did not cause vasodilation or increases in blood flow. In clinical practice, PGE_1 _derivative will be given orally or as an intravenous bolus. In the present study, PGE_1 _derivative was administrated orally and the duration of the vasodilatory effect was 90 minutes. In this experimental setting, it was difficult to investigate the duration of the effect of the PGE_1 _derivative after oral administration; however, because the half-life of this drug is 7 hours through the stomach in a rat and the increased of vasodilatation was approximately 9% at 90 minutes, we can assume that the duration of vasodilatation was more than 90 minutes in this model.

The actions of PGE_1 _are mediated primarily by the IP receptor, and include a vasodilatory effect as well as a platelet aggregation inhibition effect mediated by PGI_2_. The IP receptor is expressed in smooth muscle cells of various organs such as the aorta, coronary arteries, pulmonary arteries, and cerebral arteries, whereas no expression is found in veins [22]. However, PGE_1 _is known to dilate both arterioles and venules [6]. In addition, cyclic GMP is associated with smooth muscle relaxation, which is a different mechanism of vasodilation mediated by the IP receptor [23]. PGE_1 _also inhibits aggregation of platelets [24] and increases peripheral venous pressure [25] in experimental studies. In a clinical study, PGE_1 _administration at a low infusion rate of 0.02 μg/kg/min increased cardiac output without altering mean arterial blood pressure and blood volume [26]. In a canine model, from 3.8 to 5.6 ng/kg/min PGE_1 _intravenously did not influence systemic mean arterial pressure. Therefore, PGE_1 _may change arterial blood flow in the nerve roots due to both primary and secondary effects.

In this study, the arteries reacted to the administration of PGE_1 _derivative whereas the veins did not. However, one limitation of this study was that the changes in the diameter and blood flow in arteries and veins were observed for only 90 minutes. Another limitation of this study was that walking capacity could not be investigated before and after administration of the PGE_1 _derivative. However, in a rat model, orally administered PGE_1 _improved walking dysfunction and blood flow [16]. The increase of nerve root blood flow may improve function of the nerve root and lead to an improvement in walking capacity. According to the previous clinical reports, PGE_1 _derivative may be a potential therapeutic agent for lumbar spinal stenosis with NIC.

## Conclusion

The PGE1 derivate may have effects of vasodilation on arteries and improve blood flow of nerve roots in chronic cauda equina compression.

## Competing interests

The author(s) declare that they have no competing interests.

## Authors' contributions

All authors participated in the design of the study. MSH, BT and MSE performed the studies and drafted the manuscript. MSE performed statistical analysis. SKO and SKI participated in coordination and helped to draft the manuscript. All authors have reas and approved the final version of the manuscript.

## Pre-publication history

The pre-publication history for this paper can be accessed here:


